# Human Umbilical Cord Mesenchymal Stem Cells Inhibit Pyroptosis of Renal Tubular Epithelial Cells through miR-342-3p/Caspase1 Signaling Pathway in Diabetic Nephropathy

**DOI:** 10.1155/2023/5584894

**Published:** 2023-04-04

**Authors:** Shuo Zheng, Ke Zhang, Yaqi Zhang, Jing He, Yu Ouyang, Ruibo Lang, Chunchun Ao, Yijia Jiang, Huan Xiao, Yu Li, Mao Li, Changyong Li, Dongcheng Wu

**Affiliations:** ^1^Department of Biochemistry and Molecular Biology, Wuhan University School of Basic Medical Sciences, Wuhan, China; ^2^R&D Center, Wuhan Hamilton Biotechnology Co., Ltd, Wuhan, China; ^3^School of Life Science, Hubei University, Wuhan, China; ^4^Department of Physiology, Wuhan University School of Basic Medical Sciences, Wuhan, China; ^5^R&D Center, Guangzhou Hamilton Biotechnology Co., Ltd, Guangzhou, China

## Abstract

Diabetic nephropathy (DN) is one of the microvascular complications of diabetes. Recent studies suggest that the pyroptosis of renal tubular epithelial cell plays a critical role in DN. Currently, effective therapeutic strategies to counteract and reverse the progression of DN are lacking. Mesenchymal stem cells (MSCs) represent an attractive therapeutic tool for tissue damage and inflammation owing to their unique immunomodulatory properties. However, the underlying mechanisms remain largely unknown. In the present study, we found that human umbilical cord MSCs (UC-MSCs) can effectively ameliorate kidney damage and reduce inflammation in DN rats. Importantly, UC-MSC treatment inhibits inflammasome-mediated pyroptosis in DN. Mechanistically, we performed RNA sequencing and identified that miR-342-3p was significantly downregulated in the kidneys of DN rats. Furthermore, we found that miR-342-3p was negatively correlated with renal injury and pyroptosis in DN rats. The expression of miR-342-3p was significantly increased after UC-MSC treatment. Moreover, miR-342-3p decreased the expression of Caspase1 by targeting its 3′-UTR, which was confirmed by double-luciferase assay. Using miRNA mimic transfection, we demonstrated that UC-MSC-derived miR-342-3p inhibited pyroptosis of renal tubular epithelial cells through targeting the NLRP3/Caspase1 pathway. These findings would provide a novel intervention strategy for the use of miRNA-modified cell therapy for kidney diseases.

## 1. Introduction

Diabetic nephropathy (DN) is one of the most common microvascular complications of diabetes [1, 2] and has been a major cause of end-stage renal disease (ESRD) in patients [3]. Currently, effective therapeutic strategies to counteract and reverse the progression of DN are lacking. DN is caused by continuous increase of blood sugar, accompanied by the production of proteinuria (>0.5 g/24 h). Moreover, DN has been categorized into two stages: microalbuminuria and macroalbuminuria [3, 4]. The histopathological characteristics of DN are glomerular hyperfiltration rate, thickening of the renal tubular basement membrane, mesangial expansion, and extracellular matrix deposition, which leads to interstitial fibrosis and eventually develops into severe diffuse and nodular glomerulosclerosis of remnant glomeruli and kidney failure [5, 6].

In the classic pyroptosis pathway, the inflammasome sensors detect diverse microbial signals and activate Caspase1 through the apoptosis-associated speck-like protein containing a CARD (ASC) adaptor. Then, cleaved-Caspase1 breaks the autoinhibitory interactions between gasdermin D's (GSDMD) N-terminal and C-terminal and triggers the release of cytokines interleukin-18 (IL-18) and interleukin-1*β* (IL-1*β*). The released GSDMD-N domain binds to the plasma membrane and generates membrane pores, which results in cell swelling and eventual lysis [7]. The NLRP3 inflammasome has been implicated in the pathogenesis of chronic kidney disease, acute kidney injury, and DN [8, 9]. Recent studies indicate that inflammasome-mediated pyroptosis is involved in the development of DN. High blood sugar-induced pyroptosis is closely related to renal fibrosis, glomerulosclerosis, and renal tubular damage. Tubular epithelial cell pyroptosis is a risk factor to tubular injury in DN [10]. Xie et al. found that the lncRNA GAS5/miR-452-5p pathway reduces oxidative stress and pyroptosis in high-glucose-stimulated renal tubular cells [11]. In patients with diabetic kidney disease (DKD), the expression levels of IL-1*β*, IL-18, and NLRP3 were significantly increased in renal biopsy samples, correlated with the aggravating of albuminuria, suggesting that inflammasome may play a critical role in the progress of DN [12].

Mesenchymal stem cells (MSCs) are stromal cells with self-renewing and multiple differentiation ability, which can be isolated from various tissues, such as umbilical cord, bone marrow, and adipose tissue [13, 14]. Human umbilical cord MSCs (UC-MSCs) have advantages including low immunogenicity, noninvasive harvest procedure, and easy expansion *in vitro* [15]. Currently, the safety and efficacy of MSCs have been applied into varieties of diseases, including graft-versus-host disease, osteoarthritis, systemic lupus erythematosus, rheumatoid arthritis, diabetes, inflammatory bowel disease, and autoimmune diseases [16, 17].

MSCs can attenuate diabetic lung fibrosis via adjusting Sirt3-mediated stress responses in rats [18]. Our previous study indicated that UC-MSCs ameliorate DN by inhibiting renal inflammation, fibrosis, and apoptosis [19, 20]. However, it is still unclear whether MSCs can inhibit pyroptosis in DN.

In our study, we used a rat model of DN to explore the efficacy and mechanism of UC-MSC-based protection against renal injury in DN. We identified miR-342-3p, a downregulated miRNA in DN rat kidney, by high-throughput sequencing and uncover the beneficial effects of UC-MSCs on the treatment of DN. Mechanistically, UC-MSCs can inhibit pyroptosis of renal tubular epithelial cells through the miR-342-3p/Caspase1 signaling pathway. These findings provide novel insights into the mechanisms of DN and new strategies for the treatment of DN.

## 2. Materials and Methods

### 2.1. Animal Experiment

Male SD rats at age of 6 weeks (body weight of 200-220 g) were purchased from Hubei Provincial Center for Disease Control and Prevention (Wuhan, China). All animal procedures were approved by the Provincial Center for Food and Drug Safety Evaluation and Animal Experiment (Permit number: 202020108).

The SD rats were randomly divided into 4 groups: control group, control+UC-MSC group, diabetic nephropathy group (DN group), and DN+UC-MSC group. After one-week adaptive feeding, a DN model was induced by intraperitoneal injection of streptozotocin (STZ, S0130, Sigma-Aldrich, USA) for once (60 mg/kg dissolved in 0.1 M citrate buffer, pH 4.5). The control groups were injected an equal amount of citrate buffer. The blood glucose levels were monitored for three consecutive days, and rats with blood glucose levels ≥ 16.7 mmol/L were considered diabetic. The 24 h urine protein and urine creatinine were measured at 6 weeks after STZ injection, and 24 h urine protein ≥ 30 mg/kg was verified as DN. UC-MSCs (2 × 10^6^) in 500 *μ*L were administered via the tail vein twice at 9 weeks and 10 weeks, while the control group received the equal volume of PBS. The rats were euthanized at 11 weeks, and the kidney and blood were harvested for further analysis.

### 2.2. Renal Function

Serum creatinine and urea nitrogen levels were measured using the kit purchased from Nanjing Jiancheng Bioengineering Institute (C011-1-1, C013-1-1, Nanjing Jiancheng Bioengineering Institute, China) according to the manufacturer's instructions. The 24 h urine of each rat was collected with a metabolic cage. Urinary albumin levels were measured with the BCA kit (P0012S, Beyotime, China) according to the manufacturer's instructions. Urine creatinine levels were measured with the kit according to the procedure (C011-2-1, Nanjing Jiancheng Bioengineering Institute, China).

### 2.3. Cell Culture and Treatment

The UC-MSCs were freshly isolated from human umbilical cord tissue after deliveries in Renmin Hospital of Wuhan University (Permit number: WDRY2019-G001). UC-MSCs were isolated, amplified, and identified to meet the characteristics of MSCs using methods described previously [19]. In brief, the umbilical cord was cut into small pieces and cultured in serum-free medium (Lonza, MD, Walkersville) supplemented with serum alternatives and L-glutamine at 37°C in a 5% CO_2_ incubator. The UC-MSCs were identified by expression of a specific panel of cell surface markers (CD105^+^, CD73^+^, CD90^+^, CD34^−^, CD11b^−^, CD45^−^, CD19^−^, and HLA-DR^−^) using Human MSC Analysis Kit (562245, BD Biosciences, USA). For multipotent differentiation potential, UC-MSCs were cultured in different inducing media (6114531, 6114541, and 6114551, DAKEWE, China), respectively. The fifth generation (P5) of UC-MSCs was used in animal experiments. To obtain the UC-MSC-conditioned medium (UC-MSC-CM), the supernatant was collected after 72 h of cell culture, filtered through a 0.22 *μ*m syringe filter, and stored at -80°C.

miR-342-3p mimic and miR-342-3p inhibitor were synthesized by RiboBio. UC-MSCs were transfected using TransIntroTM EL Transfection Reagent (FT201-02, TransGen, China) according to the manufacturer's instructions.

The rat renal TECs (NRK52E) were maintained in Dulbecco's modified Eagle's medium (DMEM)/low-glucose (5.5 mmol/L) medium (C11885500BT, Gibco, USA) with 10% fetal bovine serum (FBS; 10099-141c, Gibco, USA). Cells were seeded into 6 wells with a density of 5 × 10^4^ cells/well when grown up to 80% confluence. After 24 hours, the medium was switched to DMEM/low glucose containing 2% FBS for another 12 hours. And then, these cells were exposed to media containing either low glucose (5.5 mmol/L D-glucose), high glucose (30 mmol/L D-glucose), or high glucose with 50% UC-MSC-CM for 24 h.

### 2.4. Histology, Immunofluorescence, and Immunohistochemistry

For histological analyses, the kidney tissue was cut longitudinally, fixed in 4% formaldehyde, and embedded into 5 *μ*m thick sections for hematoxylin and eosin (H&E) and periodic acid-Schiff (PAS).

For immunofluorescence staining, kidney paraffin slices were dewaxed into water. Then, the antigen was retrieved with EDTA antigen retrieval buffer (pH 8.0). After cooling, the slides were washed with PBS (pH 7.4) for 3 times. After the sections were slightly dried, a special pen was used to draw a circle around the slices and the autofluorescence quencher was added to slices for 5 minutes. BSA was dropwise added to slices and incubated. Then, slices were incubated at 4°C overnight with 1 : 100 Ly6G (1 : 200, 87048S, Cell Signaling Technology, USA) or CD11b (1 : 500, ab133357, USA), respectively. After washing with PBS for 3 times, the secondary antibody (Boster, China) was added to the slices and incubated at room temperature for 50 minutes in the dark. After washing with PBS for 3 times, DAPI staining was performed. Then, images were detected by a fluorescence microscope (Eclipse Ci-E, Nikon, Japan).

For immunohistochemistry staining, the kidney paraffin slices were dewaxed into water. Then, the antigen was retrieved with EDTA antigen retrieval buffer (pH 8.0). The slices were washed with PBS for 3 times blocked with 3% H_2_O_2_ and then blocked with bovine serum albumin BSA for 30 minutes at room temperature. Then, slices were incubated with antibody against NLRP3 (1 : 600, GB11300, China), Caspase1 (1 : 600, GB11383, China), and IL-1*β* (1 : 800, GB11113, China), which were purchased from Servicebio. After washing 3 times with PBS, the slices were incubated with CY3-conjugated anti-rabbit or anti-mouse IgG (1 : 400, Thermo, USA) or FITC-conjugated anti-mouse IgG (1 : 400, Abcam, UK). Staining was developed with DAPI to visualize the nuclei. Positive staining areas were quantified with Image-Pro Plus 6.0 software.

### 2.5. RNA Extraction and Real-Time PCR

Total RNA from the kidney or NRK52E was extracted using RNAiso Plus reagent (9109, Takara, Japan). cDNA synthesis and qRT-PCR were carried out with the PerfectStart Uni RT+qPCR kit (AUQ-01, TransGen, China) according to the manufacturer's instructions. All reactions were performed twice. The sequences of primers were as follows:

Rat NLRP3: 5′-CAGACCTCCAAGACCACGACTG-3′ and 5′-CATCCGCAGCCAATGA ACAGAG-3′

Rat IL-18: 5′-GACAAAAGAAACCCGCCTG and 5′-ACATCCTTCCATCCTTCACAG

Rat IL-1*β*: 5′-CTCACAGCAGCATCTCGACAAGAG-3′ and 5′-TCCACGGGCAAGACA TAGGTAGC-3′

Rat Caspase1: 5′-CACATGAAAGAATATGCCTGGTC-3′ and 5′-GTCCTGGGAAGAGG TAGAAAC-3′

Rat ASC: 5′-CTTAGAGACATGGGCATACAGG-3′ and 5′-CAATGAGTGCTTGCCTGT G-3′

Rat GSDMD: 5′-CACGGGACAAGGGAAAATTTC-3′ and 5′-AGGATTTTGTTTTCAGG CTGC-3′

miRNA from the kidney tissue was extracted using RNAiso for small RNA reagent (9753A, Takara, Japan). RNA was reverse transcribed using a Mir-X miRNA First-Strand Synthesis Kit (638315, Takara, Japan). Real-time PCR was performed with UltraSYBR Mixture (CW0957M, Cwbio, China).

The expression of each gene was calculated by the 2^–*ΔΔ*Ct^ method. *β*-Actin and U6 were used as the internal reference.

### 2.6. Western Blotting

Tissue or cells were lysed in RIPA lysis buffer (P0013B, Beyotime, China) with 1 mM PMSF (BL507A, Biosharp, China). After centrifugation at 12,000 rpm for 15 min, the supernatant was collected for follow-up experiment. The concentration of protein was measured by a BCA kit (P0009, Beyotime, China). Then, the protein extracts were separated on 10% SDS-PAGE gels and transferred onto polyvinylidene difluoride (PVDF) membranes (ISEQ00010, Millipore, USA). The membranes were blocked for 1 hour at room temperature in 5% milk. The primary antibody against NLRP3 (1 : 3000, NBP2-12446, Novus Biologicals, USA) was purchased from Novus Biologicals and Caspase1 (1 : 1000, 22915-1-AP, Proteintech, USA) was purchased from Proteintech. Antibody against Caspase1-p20 (1 : 1000, sc-398715, Santa Cruz Biotechnology, USA), ASC (1 : 1000, sc-514414, Santa Cruz Biotechnology, USA), GSDMD (1 : 1000, sc-393656, Santa Cruz Biotechnology, USA), and IL-1*β* (1 : 1000, sc-12742, Santa Cruz Biotechnology, USA) were purchased from Santa Cruz Biotechnology. Moreover, antibody against *β*-actin (1 : 10000, 66009-1-Ig, USA) was purchased from Proteintech. Band intensities were quantified by ImageJ software.

### 2.7. Dual-Luciferase Reporter Assay

The dual-luciferase reporter vector pmirGLO (E1330, Promega, USA) was purchased from Promega. The 3′-UTR of Caspase1 containing the conserved miR-342-3p binding sites was synthesized by Tianyi-Huayu Gene Sci-Tech Co., Ltd and cloned into pmirGLO vector. The 3′-UTR of Caspase1 containing the conserved miR-342-3p binding site mutation was synthesized and cloned into pmirGLO vector as well. The primer sequences used were as follows: for the WT, forward: 5′-CTAGCCTAAAATCCAACACTGTGTGAGCT-3′ and reverse: 5′-CTAGAGCTCACACAG TGTTGGATTTTAGG-3′ and for the mutation, forward: 5′-CTAGCCTAAAATCCAA CATCTGTGAGGCT-3′ and reverse: 5′-CTAGAGCCTCACAGATGTTGGATTTTAGG-3′. HEK293T cells were seeded in 6 wells (1 × 10^5^ per well) and cultured for 24 h before transfection. The cells were cotransfected with a mixture of 2.5 *μ*g reporter plasmids (pmirGLO-Caspase1-WT or pmirGLO-Caspase1-mut), 100 pmol miR-342-3p mimics, or negative control by TransIntroTM EL Transfection Reagent (FT201-02, TransGen, China). After 48 h, the luciferase activity was analyzed by Dual-Luciferase Assay Kit (FR201-01, TransGen, China). Renal luciferase activity was used to normalize the transfection efficiency.

### 2.8. Enzyme-Linked Immunosorbent Assay (ELISA)

The concentrations of IL-18 and IL-1*β* in DN rat kidney were measured using the ELISA kit (CSB-E04610r and CSB-E08055r, CUSABIO, China) according to the manufacturer's instructions. Cytokine levels were presented by cytokine concentration/albumin concentration.

### 2.9. miRNA Sequencing and Analysis

The kidney tissue of the DN and control groups was submitted to Majorbio Platform for RNA extraction, quantity control, and high-throughput sequencing. The data were analyzed on the online platform of Majorbio Cloud Platform.

### 2.10. Statistical Analysis

All data were presented as mean ± standard deviation (SD). Differences between groups were analyzed using an unpaired Student *t* test (for comparison between two samples) or analysis of variance (ANOVA) (for multiple comparisons). Statistical calculations were performed using GraphPad Prism. Statistical significance was accepted at *p* < 0.05.

## 3. Results

### 3.1. Characterization of UC-MSCs

**Figure 1:**
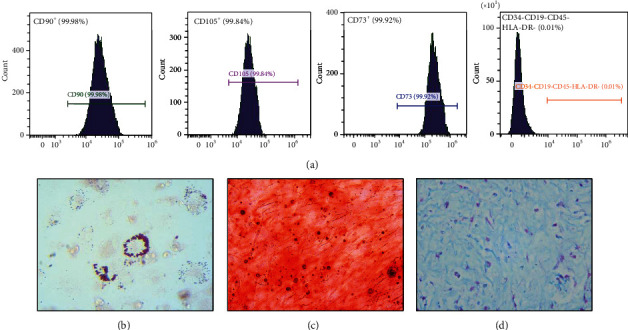
Identification of UC-MSCs. (a) Specific surface markers of P5 UC-MSCs were examined by flow cytometry. (b–d) The potency of adipogenesis, osteogenesis, and chondrogenesis of P5 UC-MSCs was confirmed by Oil Red O, Alizarin Red, and Alcian Blue, respectively.

The fifth generation (P5) of UC-MSCs was identified by flow cytometry. The proportion of positive cells (CD73^+^, CD90^+^, and CD105^+^) is more than 95%, while that of the negative cells (CD34^−^, CD11b^−^, CD45^−^, CD19^−^, and HLA-DR^−^) is less than 2% (Figure 1(a)). Then, the potency of adipogenesis, osteogenesis, and chondrogenesis was confirmed by positive staining with Oil Red O, Alizarin Red, and Alcian Blue (Figures 1(b)–1(d)).

### 3.2. UC-MSC Administration Attenuates STZ-Induced DN

**Figure 2:**
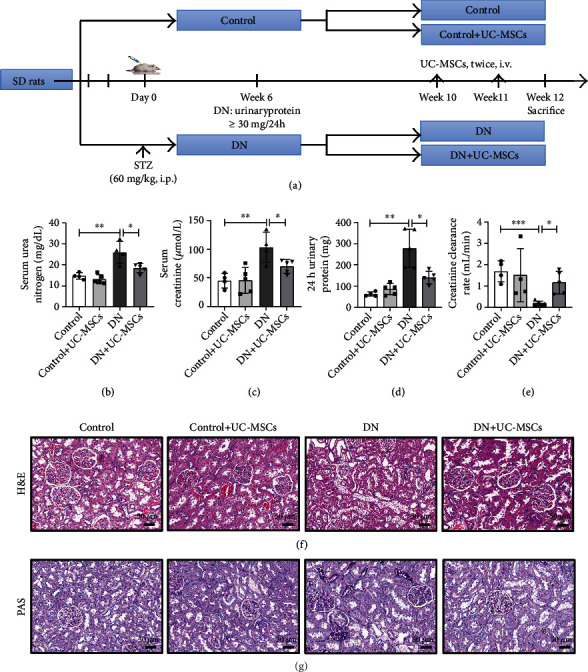
UC-MSC administration attenuates STZ-induced DN. (a) Schematic diagram of the experimental procedure. SD rats were treated with 60 mg/kg STZ or citrate buffer to induce a DN model. UC-MSCs (2 × 10^6^/500 *μ*L) were administrated twice via the tail vein after 10 weeks of STZ injection. The serum urea nitrogen (b), serum creatinine (c), 24 h urinary protein (d), and creatinine clearance rate (e) were evaluated (*n* = 4–5 for each group). Representative images of H&E staining (f) and PAS staining (g), scale bar: 50 *μ*m. Data are expressed as means ± SD. ^∗∗^*p* < 0.01 and ^∗^*p* < 0.05.

To investigate the effect of UC-MSCs on DN rats, we established a STZ-induced DN model. At 10 weeks after the model was established, the DN groups were injected with 2 × 10^6^ UC-MSCs via the tail vein, while the control group was injected with an equal volume of PBS. A detailed experimental scheme is shown in Figure 2(a). Animals were sacrificed, the kidney tissue and blood were collected at 12 weeks. We detected the biochemical indicators related to renal function such as 24 h urinary protein, serum creatinine, and serum urea nitrogen and calculated the creatinine clearance. Results indicated that the renal function of DN rats was significantly attenuated after UC-MSC treatment (Figures 2(b)–2(e)).

In addition, H&E staining showed that treatment with UC-MSCs remarkably reduced the morphological and especially ameliorated the vacuolar degeneration of renal tubular epithelial cells (Figure 2(f)). Meanwhile, PAS staining showed extracellular matrix deposition in DN rats, which was attenuated by UC-MSC treatment (Figure 2(g)). Taken together, these results suggest that UC-MSC treatment ameliorated STZ-induced renal injury.

### 3.3. UC-MSC Administration Inhibits Inflammation in DN Rats

**Figure 3:**
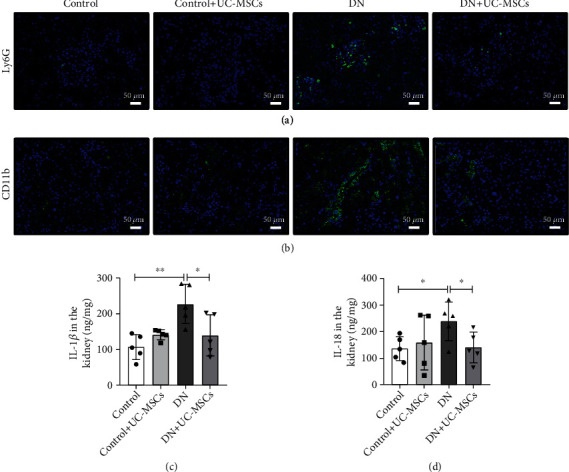
UC-MSC administration inhibits inflammation in DN rats. (a, b) Immunofluorescence staining of Ly6G^+^ neutrophils or CD11b^+^ macrophages in kidney tissues is presented (*n* = 3 for each group). Scale bar, 50 *μ*m. (c, d) ELISA analysis of IL-18 and IL-1*β* levels in kidney tissues from the control, control+UC-MSC, DN, and DN+UC-MSC groups. Data is shown as mean ± SD (*n* = 4). ANOVA was used for comparison among multiple groups. ^∗^*p* < 0.05 and ^∗∗^*p* < 0.01.

Next, we investigated the infiltration of inflammatory cells in DN, including neutrophils and macrophages. The results indicated that the numbers of Ly6G^+^ cells in UC-MSC treatment rats were significantly lower than those in the DN groups (Figure 3(a)). Furthermore, accumulation of CD11b^+^ cells was observed in DN rats compared to the control groups. In the UC-MSC treatment groups, the number of CD11b^+^ cells obviously decreased (Figure 3(b)). Then, we test the concentration of IL-1*β* and IL-18 by ELISA. Compared with the DN groups, the levels of IL-1*β* and IL-18 decreased in the UC-MSC treatment groups (Figures 3(c) and 3(d)). These findings indicate that UC-MSC administration inhibits inflammation in DN.

### 3.4. UC-MSCs Inhibit Renal Pyroptosis through the NLRP3/Caspase1 Signaling Pathway *In Vivo*

**Figure 4:**
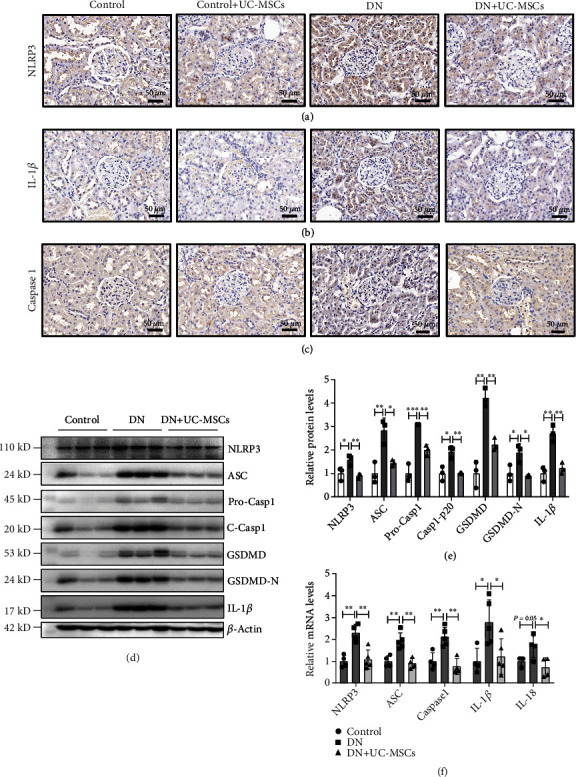
UC-MSCs inhibit renal pyroptosis through the NLRP3/Caspase1 signaling pathway *in vivo*. (a–c) Representative graphs of kidney sections with immunohistochemistry staining for NLRP3, Caspase1, and IL-1*β*. Scale bar, 50 *μ*m. (d, e) The protein levels of NLRP3, ASC, Caspase1, GSDMD, and IL-1*β* were measured by western blot in the control, DN, and DN+UC-MSC groups. (f) RT-PCR analysis of NLRP3, ASC, Caspase1, IL-1*β*, and IL-18 mRNA expressions in the control, DN, and DN+UC-MSC groups. Data are expressed as means ± SD. ANOVA was used for comparison among multiple groups. ^∗^*p* < 0.05 and ^∗∗^*p* < 0.01.

Recent studies have shown that NLRP3 inflammasome participated in many inflammatory diseases including DN [21, 22]. We wondered whether UC-MSCs can attenuate renal pyroptosis through inhibiting activation of NLRP3 inflammasome. Immunohistochemistry staining showed that UC-MSC treatment reduced the levels of NLRP3, Caspase1, and IL-1*β* in DN rats (Figures 4(a)–4(c)). Furthermore, we also observed significantly increased production of mRNA and protein of pyroptosis-related molecules in DN rats compared to the control groups, which was relieved by UC-MSC treatment (Figures 4(d)–4(f)). Taken together, UC-MSCs inhibit renal pyroptosis via NLRP3/Caspase1-mediated pyroptosis signaling.

### 3.5. miR-342-3p Is Identified as the Most Closely Related to Pyroptosis and DN

**Figure 5:**
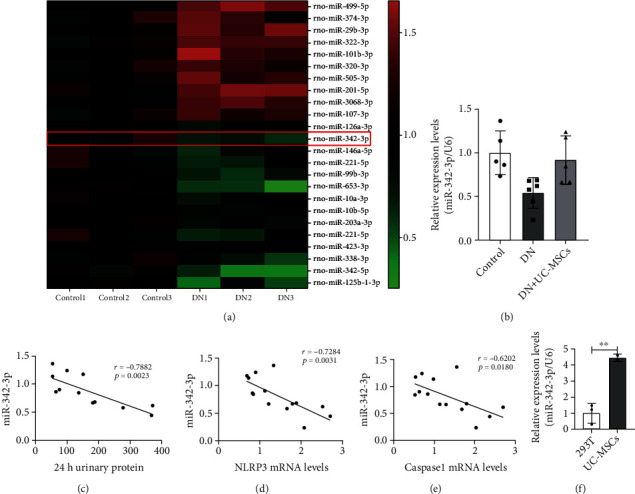
miR-342-3p is identified as the most closely related to pyroptosis and DN. (a) Heatmap of mRNA sequencing data from the control and DN groups (green, downregulated; red, upregulated); miR-342-3p was downregulated in the kidney of DN rats. (b) RT-PCR analyzed expression of miR-342-3p in the control, DN, and DN+UC-MSC groups (*n* = 4–6 rats per group). (c) Correlations of miR-342-3p expression with 24 h urinary protein in rats, *n* = 12 rats. (d, e) Correlations of miR-342-3p expression with mRNA levels of Caspase1 and NLRP3 (*n* = 11–13 rats). (f) RT-PCR analyzed expressions of miR-342-3p in HEK293T and UC-MSCs. Data are expressed as means ± SD. ANOVA was used for comparison among multiple groups. ^∗^*p* < 0.05 and ^∗∗^*p* < 0.01.

MicroRNAs (miRNAs) have a potential role in regulating the pathogenesis of several diseases, including DN [23]. To elucidate the key miRNAs involved in DN, we analyzed miRNA transcripts using RNA sequencing (RNA-seq). The heatmap analysis revealed differentially expressed miRNAs with statistical significance (FDR value < 0.05, fold change > 1) between the DN and control groups (Figure 5(a)). We validated 7 candidate miRNAs by qRT-PCR and found that miR-342-3p was identified as the most significantly downregulated miRNA in the kidney of DN rats (Figure 5(b)). Importantly, significant negative correlations of miR-342-3p with 24 h urinary protein were observed in rat urine (Figure 5(c)). Additionally, miR-342-3p expression was negatively correlated with mRNA levels of Caspase1 and NLRP3 in DN rat kidney (Figures 5(d) and 5(e)). Next, we detected the expression of miR-342-3p between HEK293T and UC-MSCs. qRT-PCR results revealed that miR-342-3p was highly expressed in UC-MSCs (Figure 5(f)). Moreover, UC-MSC treatment obviously increased miR-342-3p expression in the kidney of DN rats, as compared with untreated controls (Figure 5(b)). These results reveal the potential function and candidate biomarker attributes of miR-342-3p in DN and suggest that UC-MSC-derived miR-342-3p may play an important role in protection against DN.

### 3.6. UC-MSC-Derived miR-342-3p Inhibits NLRP3 Inflammasome Activation *In Vitro* through Targeting the 3′-UTR of Caspase1

**Figure 6:**
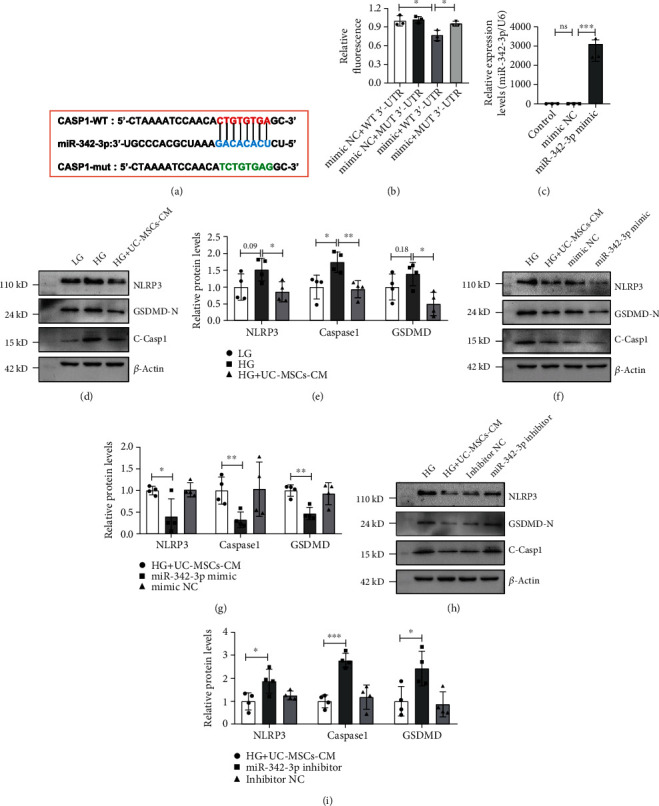
UC-MSC-derived miR-342-3p inhibits NLRP3 inflammasome activation *in vitro* through targeting the 3′-UTR of Caspase1. (a) Putative conserved binding sites of Caspase1 with miR-342-3p and the seed sequence of miR-342-3p. (b) Luciferase activity in HEK293T transfected with constructed plasmids. (c) The expression of miR-342-3p in control, NC mimic-transfected, and miR-342-3p mimic-transfected UC-MSCs. The supernatant was collected as UC-MSC-CM. (d–i) Protein expression of NLRP3, Caspase1, and GSDMD was assayed by western blot. The data were representative of the results of three independent experiments. Data are expressed as means ± SD. ANOVA was used for comparison among multiple groups. ^∗^*p* < 0.05 and ^∗∗^*p* < 0.01.

To explore the biological functions of miR-342-3p, we predicted the potential targets of miR-342-3p related to inflammasome using miRanda. Interestingly, we found that miR-342-3p has potential target sites with Caspase1 (Figure 6(a)). To evaluate the binding of miR-342-3p with Caspase1, the wide-type 3′-UTR sequence and the mutated one were cloned into pmirGLO vectors, respectively. The reporter constructs were cotransfected into HEK293T with miR-342-3p mimic or mimic NC. The dual-luciferase assay showed that miR-342-3p decreases pmirGLO-Caspase1-WT activity by binding to the target sequence. Furthermore, the inhibition of luciferase was abolished after mutation of the binding sites (Figure 6(b)).

To further determine whether miR-342-3p plays a crucial role in attenuating pyroptosis of TECs, miR-342-3p mimics and inhibitors were transfected into UC-MSCs. After 48 h of transfection, the supernatant was collected as UC-MSC-conditioned medium (UC-MSC-CM). We detected the overexpression levels of miR-342-3p using qRT-PCR assay. As expected, the expression of miR-342-3p was increased significantly (Figure 6(c)). Then, UC-MSC-CM was used to treat high-glucose-stimulated NRK52E (rat renal tubular epithelial cells). As shown in Figures 6(d) and 6(e), UC-MSC-CM treatment significantly inhibited high-glucose-induced activation of the NLRP3 inflammasome and its downstream molecules, such as cleavage of GSDMD. In addition, miR-342-3p-overexpressed CM treatment remarkably downregulated the protein levels of NLRP3, cleaved-Caspase1, and GSDMD-N compared to UC-MSC-CM treatment only (Figures 6(f) and 6(g)). In contrast, inhibition of miR-342-3p aggravated rTEC pyroptosis through upregulating protein levels of NLRP3, cleaved-Caspase1, and GSDMD-N compared to UC-MSC-CM treatment only (Figures 6(h) and 6(i)). These results demonstrate that miR-342-3p inhibited the activation of NLRP3 inflammasome through targeting the 3′-UTR of Caspase1.

## 4. Discussion

**Figure 7:**
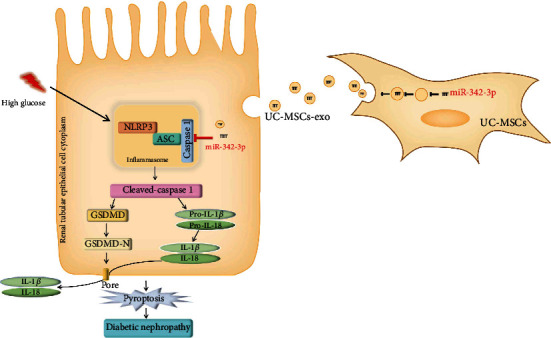
Schematic shows that UC-MSC-derived miR-342-3p attenuates pyroptosis of renal tubular epithelial cells through targeting the 3′-UTR of Caspase1 in DN.

DN has been a severe microvascular complication resulting from lesions in the renal glomeruli and tubuli, which may progress to ESRD [24]. In the present study, we found that miR-342-3p was downregulated in the DN rats and restored in the UC-MSC-treated DN rats. Moreover, miR-342-3p is negatively correlated with DN and pyroptosis and highly expressed in UC-MSCs. Using double-luciferase assay, we demonstrated that Caspase1 is the direct target of miR-342-3p. In addition, UC-MSCs prevent renal injury by releasing miR-342-3p, resulting in decreased expression of cleaved-Caspase1 and NLRP3, leading to inhibition of pyroptosis and release of IL-18 and IL-1*β* (Figure 7). These results suggest a novel intervention for pyroptosis of renal tubular epithelial cells by targeting Caspase1 in DN.

Current studies have suggested that pyroptosis-induced cell death promotes several diabetic complications, including DN [25]. Li et al. found that pyroptosis-associated proteins, including GSDMD, NLRP3, Caspase1, and IL-1*β*, were upregulated in renal tubules [26]. In HG-treated mouse podocytes, GSDMD promotes pyroptosis-regulated cell death [27]. One of the important findings in our study is that UC-MSC treatment attenuates the pyroptosis in DN. Our *in vivo* study showed that the expression of markers of NLRP3 inflammasome activation, such as Caspase1, ASC, and IL-1*β* in DN rat kidney, was significantly increased. Moreover, UC-MSC treatment notably reduced the expression of NLRP3, Caspase1, ASC, and IL-1*β*. Then, we explored the mechanism of effects of UC-MSC treatment *in vitro*. In HG-induced NRK52E cells, UC-MSC-CM significantly inhibited the expression of NLRP3, Caspase1, and ASC. Meanwhile, UC-MSC-CM reduced the release of IL-1*β* and IL-18 in the supernatant. These results provide a potential therapeutic approach of UC-MSCs to prevent the activation of inflammasome and pyroptosis in DN.

More recently, microvesicles and exosomes derived from MSCs contain cytokines, growth factors, and miRNAs [28]. miRNAs are a class of noncoding endogenous RNAs that negatively regulate gene expression through binding to the 3′-UTR of target gene [29]. To explore the miRNAs involved in DN, we performed RNA sequencing on control and DN kidney tissue and selected the differentially expressed miRNA closely related to DN, among which miR-342-3p was the top one downexpressed miRNA in DN. Recently, some studies have revealed that miR-342-3p has been reported to participate in the progression of DKD [30, 31]. In particular, Jiang et al. found that miR-342-3p suppresses renal interstitial fibrosis in DN by targeting SOX6 [31]. Our *in vivo* study showed that miR-342-3p was significantly downregulated in DN rats, whereas it was highly expressed in UC-MSCs. Importantly, we found that the miR-342-3p/Caspase1 pathway can regulate the pyroptosis of renal tubular epithelial cells in DN rats, which is consistent with the previous studies [32].

Moreover, our *in vitro* study showed that the expression of NLRP3 and Caspase1 was significantly downregulated by miR-342-3p-overexpressed UC-MSC-CM in high-glucose-induced NRK52E cells. Although it is unclear whether UC-MSC-derived miR-342-3p can also suppress renal interstitial fibrosis, our *in vivo* and *in vitro* data suggest that UC-MSC-derived miR-342-3p may be a promising strategy for the treatment of DN, through inhibiting pyroptosis of renal tubular epithelial cells by targeting the NLRP3/Caspase1 pathway.

## 5. Conclusions

In conclusion, our study identified miR-342-3p as the most closely related to pyroptosis and DN. UC-MSC-derived miR-342-3p was shown to inhibit the activation of NLRP3 inflammasome through targeting the 3′-UTR of Caspase1 in renal tubular epithelial cells, which provides a beneficial strategy for UC-MSC administration on DN. Further studies are needed to clarify whether miR-342-3p-overexpressed UC-MSCs can enhance the efficacy of renal function improvement in DN.

## Data Availability

All data generated and/or analyzed during this study are included in this published article.

## Abbreviations

DN: Diabetic nephropathy
MSCs: Mesenchymal stem cells
UC-MSCs: Human umbilical cord MSCs
ESRD: End-stage renal disease
ASC: Apoptosis-associated speck-like protein containing a CARD
GSDMD: Gasdermin D
IL-18: Interleukin-18
IL-1*β*: Interleukin-1*β*
DKD: Diabetic kidney disease
STZ: Streptozotocin
DMEM: Dulbecco's modified Eagle's medium
H&E: Hematoxylin and eosin
PAS: Periodic acid-Schiff
PVDF: Polyvinylidene difluoride
ELISA: Enzyme-linked immunosorbent assay
SD: Standard deviation
miRNAs: MicroRNAs
RNA-seq: RNA sequencing
UC-MSC-CM: UC-MSC-conditioned medium.
